# A validated geomechanical model for the strike-slip restraining bend in Lebanon

**DOI:** 10.1038/s41598-022-24718-0

**Published:** 2022-11-22

**Authors:** Jakub Fedorik, Francesco E. Maesano, Abdulkader M. Afifi

**Affiliations:** 1 King Abdullah University of Science and Technology (KAUST), Thuwal, Saudi Arabia; 2 Istituto Nazionale Di Geofisica E Vulcanologia (INGV), Roma, Italy

**Keywords:** Geodynamics, Tectonics

## Abstract

Most of the methodologies used to validate complex strike-slip structures mainly rely on comparison with other well-known geological features or analogue laboratory models. This study adopts an approach based on the boundary element method at the regional scale to test the structural interpretation of a complex transpressional mountain range. Lebanon restraining bend represents the most prominent topographic transpressional feature along the Dead Sea Transform (DST). It consists of two mountain ranges: the Mount Lebanon and the Anti-Lebanon ranges. We built a 3D geometrical model of the fault surfaces based on previously studied natural examples, structural maps, satellite images, DEM interpretation and experimental analogue models of restraining bend or transpressional structures. Using a boundary element method, we modelled fault deformation response to the regional stress field. The simulation accurately predicts the shape and magnitude of positive and negative topographic changes and fault slip directions throughout the study area. We propose an original approach, which uses implementation of well-known fault geometries, surface and subsurface data, for structural validation in the complex strike-slip domain. Our results, validated by structural evidences, highlight that various structural styles lead to formation of Mt. Lebanon, Anti-Lebanon and Palmyrides structures. Furthermore, this simulation supports the hypothesis that the restraining bend of the DST formed in the widespread crustal weakness zone developed in the Late Jurassic to Early Createceous. We also propose recent Neogene tectonic evolution of the region based on our modelling and integrated with published U/Pb dating of fault zones and tectonostratigraphic evidence.

## Introduction

Transcurrent faulting affects many parts of the Earth's lithosphere and occasionally results in prominent topographic expressions^1–4^. Strike-slip faults pose major challenges in understanding their structural setting and evolution due to closely-spaced fault planes with sub-vertical attitudes and non-planar geometries along strike. Transpressional systems may extend to deep crustal levels as nearly vertical structures. Such geometry allows the wrench component of movement to have significant uplift rates due to colliding blocks^5^.

Scaled analogue modelling has proved to be a powerful tool in understanding the geometries and kinematics of complex 3D structures in restraining bend structures^6,7^. These authors compare results from analogue models to several natural cases showing important similarities between both examples. In particular, all structures are characterised by uplifted pop-up structures with faults converging with increasing depth.

Many applications, such as fault-based seismic hazard assessment, are influenced by the knowledge of fault 3D geometries at depth^8,9^, but information on the down-dip fault geometries are often lacking, and indirect while robust estimates are needed.

The Lebanon restraining bend (LRB) is one of the best examples of transpressive structures worldwide. LRB also represents a significant source of seismic hazard for the region hosting several historically active faults^10,11^. Besides the importance of LRB in the tectonic evolution of the area and its present-day activity, the knowledge of its subsurface geometry is poor. In particular, seismic reflection surveys imaged and constrained only its offshore segments^10,12^. The onshore deformation was studied thanks to surface geological methods^11,13–15^, while passive seismic methods, gravity, magnetic and seismic reflection surveys in the area are sparse^16^ or lacking.

By integrating the limited subsurface information with surface fault traces and structural styles observed in laboratory analogue models, we propose a 3D subsurface fault model for the entire LRB area. We tested the validity of the structural model by comparing the faults response to regional stress with the present-day kinematics and topography through a boundary element method (BEM) numerical model^17^. This technique was performed successfully in other tectonic settings but on a much smaller scale^18–20^. This research provides new insights into the structural complexity of the LRB. Based on tectonostratigraphic^16,21^ and U/Pb dating evidence^22^, and our results, we propose a potential tectonic evolution for this area. The strategy adopted in this study may provide a valuable tool for integrating surface data with reliable crustal 3D fault models to be included in seismic hazard studies where subsurface data are missing.

## Structural setting

**Figure 1:**
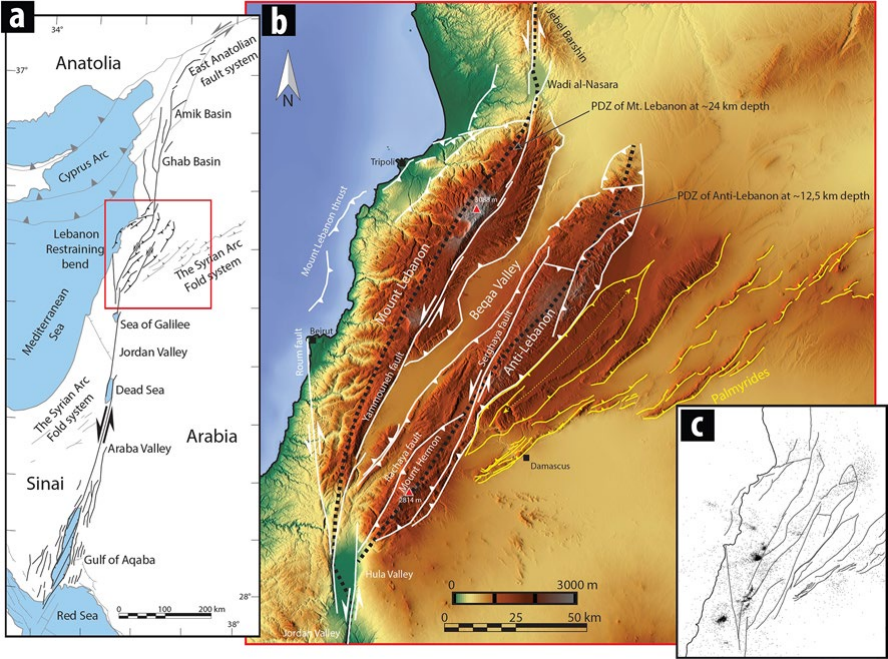
Structural map of Lebanon Restraining bend. (**a**) Different tectonic structural styles along the Dead Sea Transform: (1) Inactive distributed shear along the onshore margins of the Gulf of Aqaba, and southward increasing transtension forming the gulf; (2) pure strike-slip deformation in the Araba and Jordan valleys; with three step-over basins in the Dead Sea, Sea of Galilee and Hula Valley; (3) a restraining bend and transpressional structures along the LRB; and (4) transtensional structures within the Ghab and Amik basins. (**b**) 90 m DEM of the studied area (red square in **a**). The white lines are the main faults of the LRB. The yellow lines are thrust faults along the Palmyride fold belt. Map obtained from maps-for-free.com. (**c**) Seismicity between 2006 and 2018 as recorded by GRAL. Data provided by CNRS Lebanon—National Center for Geophysical Research (http://www.cnrs.edu.lb/english/research/researchcenters/national-center-for-geophysics---download).

The Dead Sea Transform (DST) is a 1000-km-long fault system that connects the Sinai triple junction to the East Anatolian fault and separates the Sinai microplate from the Arabian plate (Fig. 1). DST strikes N0°–N10°E producing a mostly negative topography in the Gulf of Aqaba, the Araba Valley, the Dead Sea, the Jordan Valley, the Hula, and the Ghab basin (Fig. 1). The LRB segment strikes ~ N30°E and creates a prominent positive topography, reaching an elevation of 3088 m in Mount Lebanon range.

The Yammouneh fault is the main strike-slip fault of the DST in Lebanon, and is the only connection between the southern and northern segments of the DST. It produces the highest elevation change in Lebanon, with mean Late Pleistocene slip rates of 5.1 ± 1.3 mm·a^−1^ based on the offset of 25-ka-old alluvial fans^23^. West of the Yammouneh fault, the Mt. Lebanon range dips gently to the NW and is bounded offshore by the Mount Lebanon thrust^13^. This curved thrust is connected to the Yammouneh fault by the Roum fault to the south and by a set of minor faults east of Tripoli (Fig. 1b). Unroofed Jurassic units along the entire length of the Mt. Lebanon range suggests laterally constant uplift (Supplementary Fig. 1).

The Anti-Lebanon range length is similar to the Mount Lebanon range, but is narrower. The central part of the Anti-Lebanon range, where limited erosion occurred^24^, forms a relatively flat topography underlain by Cretaceous strata whose dip progressively increases towards the edges of the range. To the south, the Anti-Lebanon range culminates at 2814 m (Mount Hermon). The Rachaya fault bounds the Anti-Lebanon range to the southwest and links with the Serghaya fault, whose slip-rate during Holocene is 1.4 ± 0.2 mm·a^−1^ based on offset buried channels^25^. The Serghaya fault cross-cut most of the Anti-Lebanon range from SE to NW. The faults recognised at the northern end of the Anti-Lebanon range do not link with the DST.

The Beqaa valley is an elongated basin located between the Mt. Lebanon and Anti-Lebanon ranges. Gravity data suggest that the Beqaa valley is filled by up to 9 km^26^ of lacustrine and continental sediments with the deepest part in the central area of the valley^27^. Review of available seismic data show no sedimentary growth related to LRB tectonic activity since the Middle Miocene, while Early Miocene layers onlap both sides of a small anticlinal structure in the central part of the Beqaa valley^16^. Oligocene layers show constant thickness across above the mentioned anticlinal structure^16^.

East of the LRB, the Palmyrides fold belt (also known as the Syrian Arc Fold belt) accommodates internal deformation within the Arabian plate^28^ reactivating inherited normal faults (e.g. ^29,30^). Evidence for Triassic rifting and regional thinning of the crust exists in southern Israel^31^ and along the Palmyrides^32^, where grabens formed as a response to the NW–SE extension. At the end of the Jurassic and the beginning of the Cretaceous (ca. 140–110 Ma), a second episode of extension likely led to seafloor spreading in the Levant basin^33,34^. Seismic reflection data^35,36^ and drilling records show the SW Palmyrides to have been controlled by NW-dipping late Paleozoic and Mesozoic normal faults that were structurally inverted in the Neogene. Several tectonic reactivations within the Syrian Arc Fold System suggest rheological weakness within the otherwise stable Arabian and Sinai plates^29^. The amount of deformation is higher in the southern area of the Anti-Lebanon (closest to the Arabia—Sinai plate boundary) and gradually decreases eastwards towards the interior of the Arabian Plate. The strike of faults in the Palmyrides is approximately N46°E, the Anti-Lebanon range strikes N38°E, while Mt. Lebanon deviates counterclockwise to N27°E.

The timing of deformation of the Anti-Lebanon and Mt. Lebanon is poorly constrained. Fault-related calcite precipitates south of Mount Hermon were dated at 17.1 ± 0.3 Ma^22^, indicating that the DST was active during the Middle Miocene. On the other hand, seismic profiles offshore Lebanon^21^ clearly show the offset of upper Miocene units by the Mount Lebanon thrust (Fig. 1), which became active during the Messinian. This important age difference suggests non-simultaneous inception of deformation along the two ranges of LRB.

Measured residual GPS velocities^37^ highlight a marked slip-rate variability along the DSFs, with higher slip-rates in the southern sector compared to the northern one. The central sector, locus of the LRB, is characterised by velocities of 5.0 ± 1.1 mm·a^-1^ for the Yammouneh fault (e.g.^38–40^), decreasing to 1.4 ± 0.2 mm·a^-1^ along the Serghaya fault (e.g.^25,40^). The active regional shortening across the Palmyrides is estimated to be less than 1 mm·a^-1^^41^.

## 3D model construction

### Fault mapping

The Restraining Bends of Lebanon are the most prominent features along the DST, where the first-order analysis on digital elevation models (DEM^42^) and satellite images can reveal major fault lineaments and limited folding of associated sedimentary units. Our interpretation was integrated with published geological maps onshore (^11,15,16,21,27,43^, Supplementary Fig. 1).

**Figure 2:**
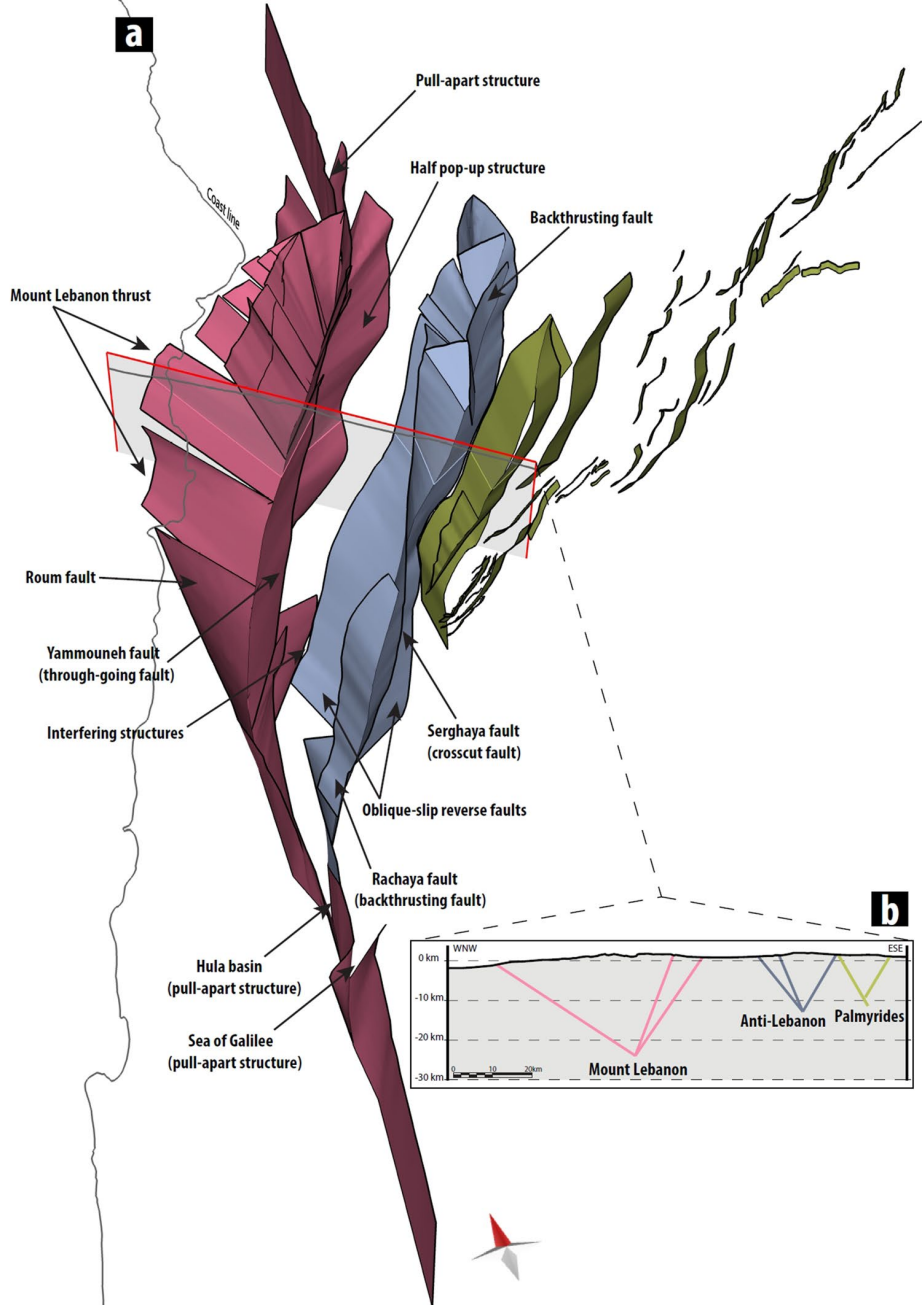
Model construction. (**a**) Three-dimensional (3D) northward view of Mt. Lebanon faults (in pink), Anti-Lebanon faults (in blue) and Palmyrides (in yellow), no vertical exaggeration. (**b**) WNW-ESE 115 km long section displaying intersecting faults of the Lebanon Restraining bend and the Palmyrides. Variable depth of PDZs were determined based on fault geometry.

Fault geometries vary significantly from Mount Lebanon to the Anti-Lebanon range. The Beqaa valley separates Mount Lebanon from Anti-Lebanon, supporting the hypothesis of two isolated mountain ranges with two different principal displacement zones (PDZ). It is worth mentioning that the southern corner of the Beqaa valley, where the two ranges meet, shows interfering structures^44^ with juxtaposed oppositely-dipping beds (^15^, Fig. 2). For these reasons, two different structural styles were selected for the subsurface conceptual fault model of the two ranges. Folding of sedimentary units is observed in several locations (e.g. NE of Anti-Lebanon, S side of Mount Hermon or fault bend folding in Palmyrides), but associated deformation is smaller compared to fault related deformation with several kms of offset. Given the characteristics of the boundary element method adopted for numerical modelling, we simplify our structural interpretation including only faults within it. Full 3D model of 88 faults of the Restraining Bends of Lebanon and Palmyrides is available in supplementary material.

### Anti-Lebanon

The mean strike of Anti-Lebanon is N38°. We based our 3D fault model (Fig. 2) on several analogue models of transpressional features along the straight plate boundary (velocity discontinuity)^45,46^. These models show elongated structures creating positive elevation changes bounded by two oblique-slip reverse faults connecting at depth to the PDZ. Sub-vertical strike-slip faults crosscut the models with more significant deformation^47,48^ (Supplementary Fig. 2b). In these models, the typical dip angle of the oblique-slip reverse faults is greater than 30 degrees. Analogue models of normal faults reactivated in transpression show steeper dip angles (35–60 degrees) for the reactivated structures^49^.

For the Anti-Lebanon we chose dips of 45 degrees for the NW-dipping faults and 60 degrees for the SE-dipping oblique-slip reverse faults, where the amount of deformation is much higher due to emplacement of back-thrusts (e.g. Rachaya fault) that form Mount Hermon. With the implementation of higher angles for the Anti-Lebanon range, we hypothesise the pre-existing normal faults created by the Syrian Arc belt have influenced the formation of the Anti-Lebanon. The subsurface intersection of bounding oblique-slip reverse faults creates a PDZ for Anti-Lebanon to which we connected a sub-vertical crosscutting fault (e.g. Serghaya fault).

### Mount Lebanon

The mean strike of Mt. Lebanon is N27°. The deformation is dominated by: a central through-going Yammouneh fault, a curved offshore Mount Lebanon thrust in the north, and a southern half pop-up structure (Fig. 2). Faults in the analogue model^50^ representing Mount Lebanon thrust show very smooth linkage (e.g. Roum fault) to central through-going fault and distributed linkage on the other side (e.g. faults E of Tripoli). In the central area of Mount Lebanon, we assumed ~ 75 degrees dip towards the NW for the Yammouneh fault and ~ 45 degrees dip towards the SE for the Mount Lebanon thrust. Similar dip values for the 3D fault pattern were observed in the clay model of the restraining bend^51^ (Supplementary Fig. 2a), while^29^ also interpret faults of Mount Lebanon as converging faults with increasing depth. The intersection between the Yammouneh fault and the Mount Lebanon thrust creates a PDZ for Mount Lebanon range, which corresponds to a plate boundary between the Arabian and the Sinai tectonic plates. The seismic line visualising subsurface west of Yammouneh fault^16^ highlights a fairly continuous dip of picked reflectors with only minor faults and no associated folding. Half pop-up structures connect to the PDZ, and therefore these faults are dipping towards the NW. Even though we consider that the Roum fault, and several faults mapped east of Tripoli are part of the oblique Mount Lebanon thrust, we preserved their segmentation in the 3D fault model.

### Palmyrides and pull-apart basins

At the southern and northern tip of the Yammouneh fault, two pull-apart basins are observed, which have a simple structure consisting of left stepping faults at the surface that connect at depth to the PDZ (e.g.^7,52,53^).The southern pull-apart basin (Hula Valley) has a more elongated structure, which means that the overlay of stepping faults is higher than in the northern basin (Wadi al-Nasara).

The SE side of Anti-Lebanon is positioned next to reactivated structures of the Palmyrides. According to^54^, folds created during structural inversion and shortening result in fault-propagation folding. Faults reaching the surface were extrapolated to a 60 degrees dip, and their depth was defined proportionally to the amount of reactivation observed at the surface.

## Results of numerical models

According to stress orientation data across the LRB and Palmyrides (Supplementary Fig. 3), we performed seven numerical models with varying orientations of the principal horizontal stress (σ1), ranging from N120–N180 (Supplementary Fig. 4). The overall numerical best fit models are for σ1 oriented N160–N170 (Table 1). Nonetheless, assuming a σ1 orientation of N140 we obtained a better result in reproducing the general shape of the LRB, in agreement with the stress rotation observed in this area (Supplementary Fig. 3 and Methods section). For this reason, in the following sections, we discuss the N140 model results for fault displacement and vertical changes.

**Table 1:** List of numerical models with variable σ1 ranging from N120 to N180. Best fit models are for σ1 oriented N160-N170. Selected model with σ1 oriented N140 (in bold) reproducing various structural features of the LRB was selected for this study.

Model	Residual mean	Residual stdev	Residual sum squares
N120	0.103	0.120	2548.0
N130	0.100	0.115	2323.7
**N140**	**0.097**	**0.109**	**2100.5**
N150	0.094	0.103	1866.4
N160	0.093	0.099	1746.7
N170	0.097	0.098	1703.2
N180	0.104	0.101	1799.0

### Fault displacement

**Figure 3:**
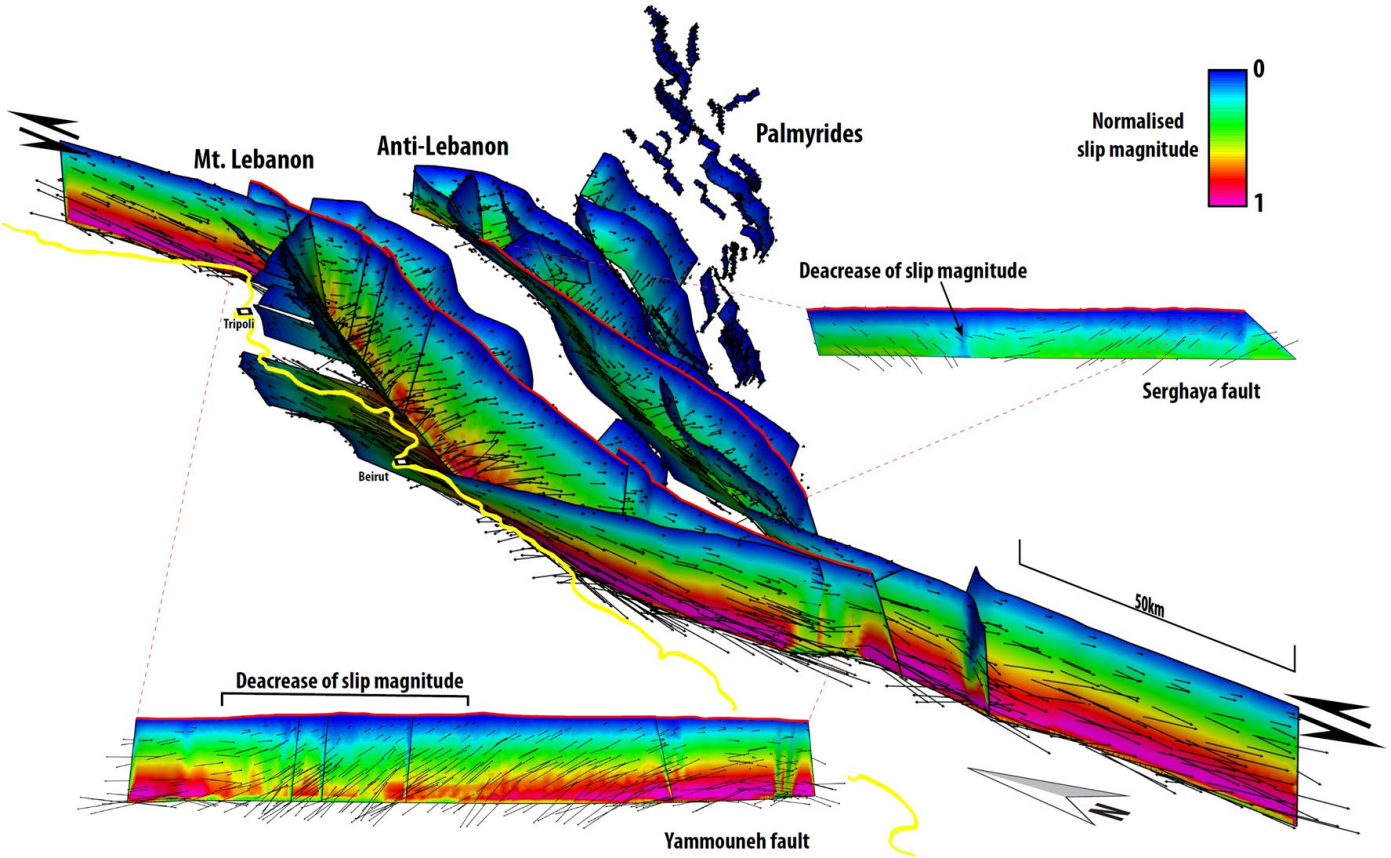
Slip magnitude. Three-dimensional (3D) Eastward view of Lebanon Restraining bend and Palmyrides with normalised slip magnitude plot. Black vectors represent orientation of displacement along all faults. Yellow line shows the coastline. Yammouneh and Serghaya are also presented in the side view. Serghaya fault slip magnitude decreases in the area where fault is sub-vertical. Yammouneh fault slip magnitude decreases dominantly due to a change of the strike of the fault. Northern and Southern parts of the Yammouneh fault are oblique to σ1, while its central part is almost orthogonal.

Our numerical model contains 88 fault surfaces. The numerical model produced the same slip directions observed in the field for all the principal faults (Fig. 3). The Yammouneh fault produces a left-lateral offset with important reverse dip-slip component along the restraining bend. The Roum fault and other NS trending faults produce a dominantly pure left-lateral strike-slip component, due to their favourable orientation. All faults whose orientation is perpendicular to the principal horizontal stress are reactivated with a dominantly reverse dip-slip component (e.g. offshore Mount Lebanon thrust; oblique-slip reverse faults bounding Anti-Lebanon). The Serghaya fault with its helicoidal shape generates a pure strike-slip component in its middle, while in the area of Mount Hermon it produces an oblique displacement of the hanging-wall towards the SE. Its northern part produces the opposite slip and displacement of the hanging wall towards NW. In general, we observed an increase of slip magnitude with an increasing depth of faults (Fig. 3). According to the present-day GPS velocity field^37^, we observe the highest slip values along the Yammouneh and Roum faults. We note that slip magnitude decreases along the Yammouneh fault where the fault strike is almost orthogonal to σ1. Segmentation of the Yammouneh fault has minimal impact on slip magnitude variation, whereas fault intersections produce a decrease in the slip observed on both faults in the intersecting area.

### Model validation

**Figure 4:**
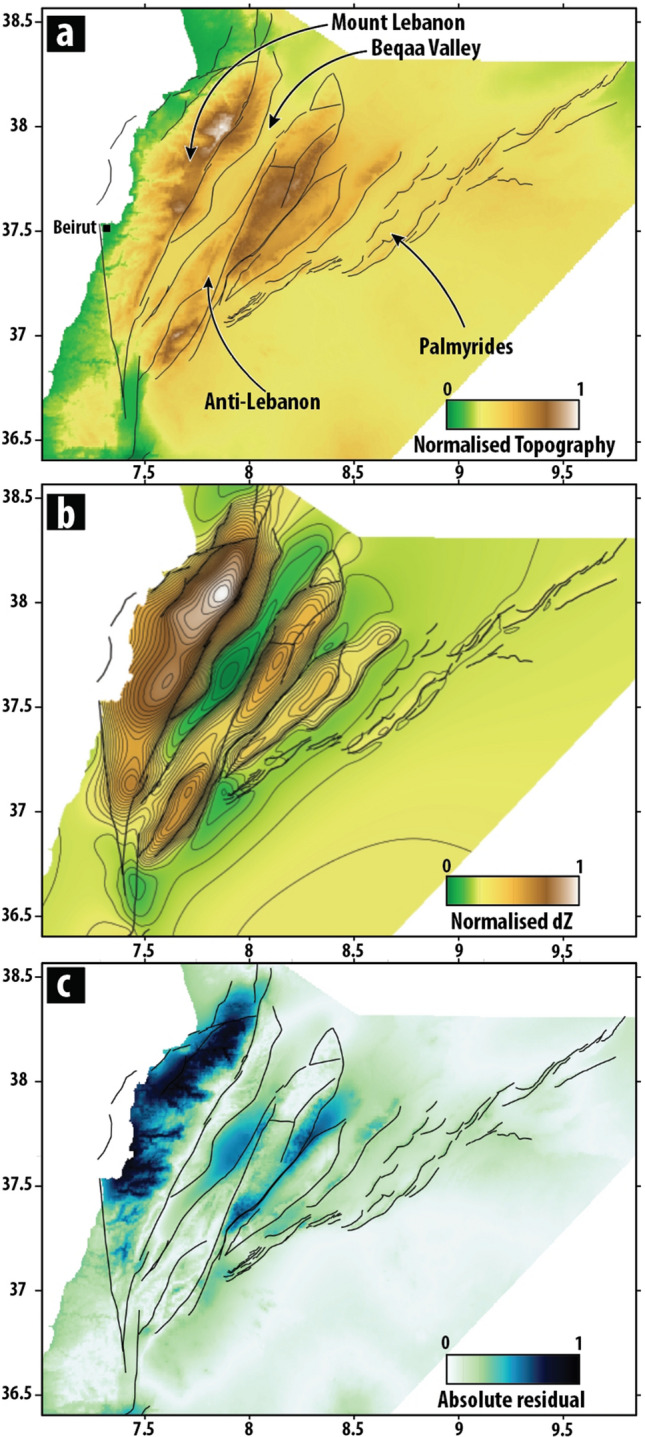
Model results. **(a**) Normalised DEM and fault traces of LRB used in the numerical model. Main structural features are listed. (**b**) Normalised vertical displacement (dZ) produced by our model with a σ1 value of N140. Results of our model with σ 1 ranging from N120-N180 are shown in Supplementary Information. (**c**) Absolute residual highlighting the main discrepancies between the normalised topography and normalised dZ.

To validate the numerical model, we compared the normalised topography of the area (Fig. 4a) to the normalised elevation produced by the models (displacement in Z direction, Fig. 4b). This comparison is viable since LRB is the only segment along the whole DST that produces significant positive elevation changes. The highest elevation change produced in our numerical model is located along the Yammouneh fault, while the highest point of the model itself is in the area of Qurnat as Sawda' which is the peak of Mt. Lebanon (Fig. 1). Numerical model produces an asymmetrical anticline between Yammouneh fault and Mount Lebanon thrust with the hinge zone in a near proximity of Yammouneh fault. The NW limb of this anticline dips towards Mount Lebanon thrust. Data from^16^ confirm these results as vertical change across Yammouneh fault is 5 km and asymmetrical anticline also observed in seismic section. Numerical model also creates half pop-up structures, observed in the same place on the SE side of Yammouneh fault.

In our simulation, a positive elevation change in Anti-Lebanon is also observed, peaking in the vicinity of Mt Hermon and central area of Anti-Lebanon. Along the Palmyrides, away from the Anti-Lebanon range, a positive elevation change of lower amplitude is produced. Step over nature of major faults in southern tip of LRB create localised depression in the area of pull-apart Hula Basin.

The absolute residual map (Fig. 4c) highlights areas where the topography produced by our numerical model differs from the existing topography. Except for the zone close to the Yammouneh and adjacent SE faults, the model produces higher topography along the Mt. Lebanon range. Due to relatively high erosion rates, discrepancies between the numerical model and elevation model are also observed in Mt. Lebanon range where Miocene, Paleocene and Cretaceous units are completely or partially eroded and Jurassic units are exposed.

Geological processes such as erosion and sedimentation are not replicated in our model, which could explain the discrepancies observed in the Beqaa valley and between Anti-Lebanon range and Palmyrides. Average thickness of stratigraphic units that are preserved in Beqaa valley is 2500 m for the Miocene-Paleocene and 2100 m for the Cretaceous^16^. The relative low created in the numerical model in correspondence of the Beqaa valley may be interpreted as the accommodation space for these sediments. Similarly, between Anti-Lebanon and the Palmyrides structure our model under-performs as it creates a narrow and elongated basin between two highs. As a first-order approximation for our model, we assigned all faults the same rheological parameters (Supplementary table 1) and we considered that these faults fully accommodate the total displacement. Also, the variability of stress orientation within the study area (Supplementary Fig. 5) impacts the results since we assumed a uniform orientation for each model. Lastly, the work budget and efficiency of fault systems^55^ of both, Mt. Lebanon and Anti-Lebanon, is not known while we compare it to a fully developed faults system in our numerical model. Overall, the main differences between the observed topography and our model result from these simplifications and from the purely elastic approximation of the numerical method.

## Synthesis and outlook

Properly selected structural styles are a crucial criterion for constructing 3D fault geometries. A well-established method (e.g. 2D fault restoration) to validate a given structural interpretation does not exist in the strike-slip domain. Hence validating a conceptual model, such as the one presented in this paper, can enhance our knowledge of the subsurface producing a significant improvement in various applications where 3D fault geometries are needed, such as seismic hazard assessment^8^.

**Figure 5:**
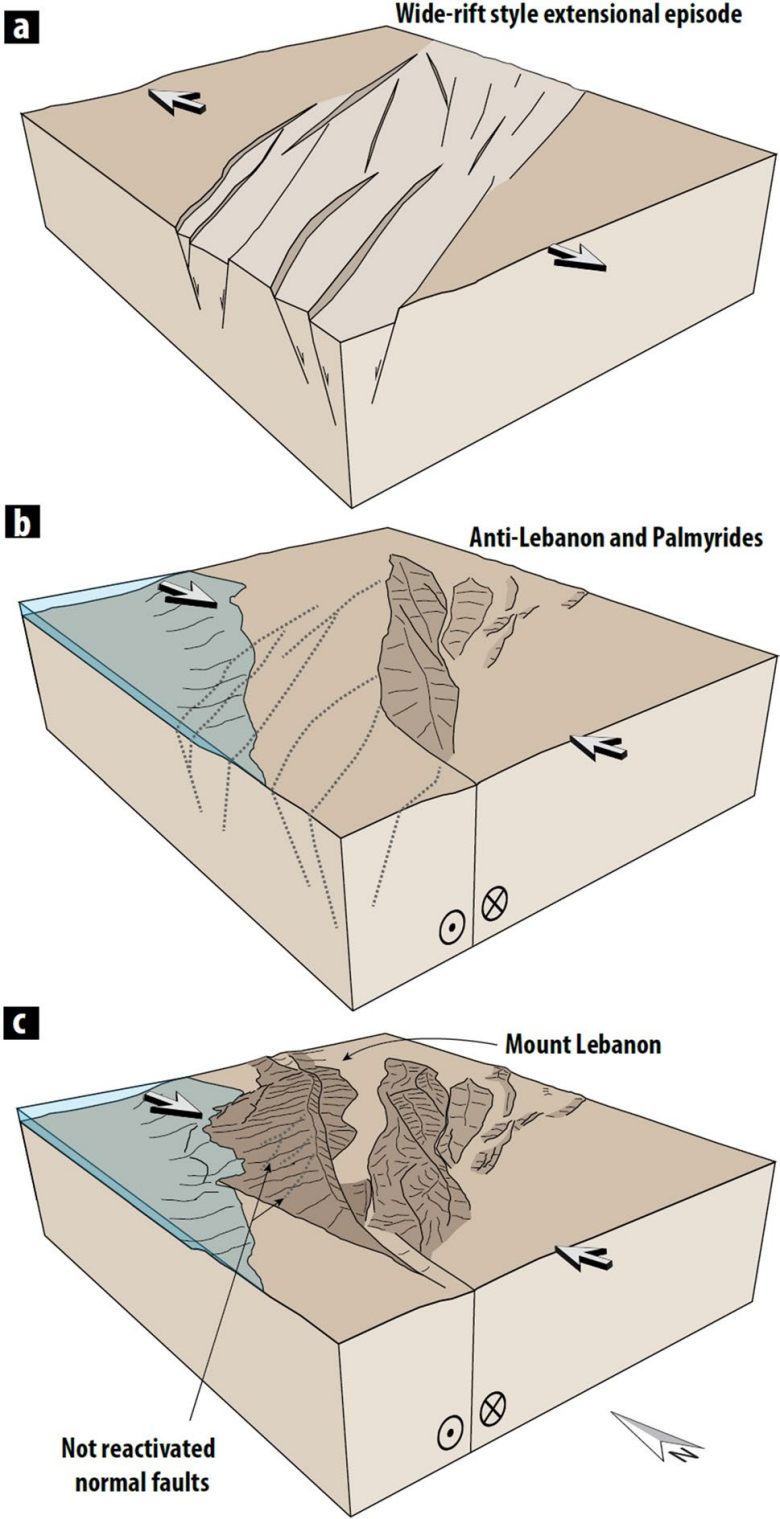
Tectonic evolution of the Lebanon Restraining Bend. (**a)** Late Jurassic to Early Cretaceous rifting produced WNW-ESE-trending basin systems^27^. (**b**) Northward propagation of DST and partial reactivation of rheological weakness such as rift faults (17 Ma^22^), resulted in the initiation of Anti-Lebanon and Palmyrides uplift. **(c**) From mid-late Miocene (~ 10 Ma to present), most of the deformation switches to Mt. Lebanon, while moderate uplift of Anti-Lebanon continues. Large white arrows represent plate-driven regional tectonic stress fields.

Two different tectonic styles observed in analogue models were used for the construction of Lebanon Restraining bend 3D fault geometry. Mt. Lebanon has identical surface expression as experiments from physical models^51,56^. A recent model^50^ shows a detailed evolution of the fault system in restraining bend, including curved thrust (e.g. Mount Lebanon thrust) nucleating with a single fault trace (e.g. Roum fault to the south) and distributed linkage on the other end of the curved thrust (e.g. faults in the area of Tripoli). For the construction of Anti-Lebanon, geometries from transpressional models^47^ were implemented. Fault response model fits well with the observed fault displacement and topography, highlighting the accuracy of this new conceptual structural model for the Lebanon Restraining bend. Our structural model confirms that the kink along the Dead Sea Transform—Lebanon Restraining bend, formed likely due the rheological weakness known as the Syrian Arc belt (Fig. 5a). We suggest that Anti-Lebanon and Palmyrides structures reactivated pre-existing normal faults (Fig. 5b). However, the Dead Sea Transform propagation, which started in the south, did not continue towards the north through the Anti-Lebanon structures but rather towards a new branch that formed to the west (Mt. Lebanon; Fig. 5c). While Anti-Lebanon started to form during early-Miocene by the reactivation along Jurassic—Cretaceous rift faults^22^, the displacement along the Mt Lebanon Thrust during mid to Late Miocene^21^ marks the early initiation of Mt Lebanon uplift. Conversely, Mt. Lebanon faults have an oblique orientation to the pre-existing normal faults which were not reactivated.

## Methods

To model the deformation associated with the LRB we used a three-dimensional boundary-element method (BEM), incorporated in Fault Response Modelling of MOVE software. Fault Response Modelling relies on the elastic dislocation theory applied on triangular elements^57^ to deform one or multiple observation grids. A complete description of the theoretical aspect regarding the boundary-element method is described in^17^.

The three-dimensional fault surfaces are modelled as meshes constituted by discrete triangles with 1000 m sampling and of constant slip and zero opening located within a linear-elastic and homogeneous material. The use of triangular meshes in BEM models allow for modelling the complex fault network of LRB where complex fault curvature, overlap and overstepping are observed as already done in similar cases (e.g., southern California^18^; southern Italy^58^).

We made some regional assumptions to constrain the elastic parameters of the model. Since seismicity is distributed within all the crust thickness down to the Moho (Supplementary Fig. 3), we assumed constant Young modulus of 40 GPa and Poisson ratio of 0.25 according to average crustal rigidity^59,60^.

The numerical model reproduces the elastic component of the deformation, thus the visco-elastic behaviour of the crust in the long-term is not incorporated in the model. We assume that the lithospheric deformation and the direction of plate motion are related to a stable stress field^61^. Selection of the regional stress driven model was made due to the large area affected by deformation. Recent numerical models of^59^ and^62^ used plate driven approach, where deformation is concentrated along the plate boundary. Our model also includes faults at significant distances from the plate boundary therefore using a model driven by regional stresses is more suitable.

Information on the stress field orientation is mainly based on published stress data and GPS velocities (World Stress Map^41,63–65^). Generally, all agree on a maximum horizontal stress oriented indicating a maximum horizontal stress oriented between N160 and N170 in the stable Arabia region between the Sinai Triple Junction and the East Anatolian fault which acts as σ1 controlling the dominant left lateral strike slip behaviour of the DST. This dominant trend is confirmed by geodetic measurements^41,63^. Near the DST, where stress data are few and sparse, major orientation variations are observed with a maximum horizontal stress oriented N130 in the Mount Lebanon range. Among the various analyses based on instrumental seismicity^41^, inversion of focal mechanisms shows significant deviation from the dominant stress of the area for the LRB, highlighting a coexistence of both strike-slip and normal faulting. Nonetheless, since our modelling focuses on the long-term deformation of the area, we consider maximum horizontal compressive stress and strike-slip tectonic regime, following the general motion direction of the Arabian Plate. Thus we explored the effect of simulated stress conditions in the LRB by applying a regional stress with horizontal σ1 ranging from N120E to NS. We therefore imposed a triaxial remote stress boundary condition to the model by assuming vertical σ2 and consistent with the lithostatic load for an average rock density of 2.6 g/cm^3^. Our reference level and observation grid are imposed at an elevation of 750 m above sea level, corresponding to the average elevation of the area.

We computed a pressure profile at a depth of 5.75 km assuming a linear gradient of the principal stresses and hydrostatic pore pressure with depth. Since there are no in-situ constraints regarding the stress magnitude at depth, we rely on available theoretical models as follow:

We imposed a stress shape ϕ = (σ2 − σ3)/(σ1 − σ3)^66,67^ of 0.25 which can be considered representative for transpressive tectonic settings^68^, and we assumed a rock friction coefficient (μ) of 0.6^69^.

Assuming that the stress magnitude in the crust cannot exceed the frictional strength of pre-existing discontinuities, we adopted the formulation proposed by^70^ and^71^, by imposing that the ratio between the maximum (σ1) and minimum (σ3) principal effective stresses is defined by:

$$\frac{{\sigma }_{1}}{{\sigma }_{3}}={\left[\sqrt{{\mu }^{2}+1}+\mu \right]}^{2}=3.12$$

Knowing the vertical stress σ2 and the assumed value for Φ, we can derive an upper boundary estimation for the maximum and minimum effective stress magnitude at a depth of 5.75 km following the formulation proposed in^72^. The input parameters of the numerical model are reported in supplementary table 1.

The regional stress field represents the boundary condition for predicting the displacement vector on each triangular element of the faults^17^. The other boundary condition is that there is no displacement normal to each fault triangle (no opening component).

Once the fault geometries were constrained by comparison with analogue models and geomorphological analysis, the results of the numerical model were focused on comparing alternative uplift/subsidence features on the observation grid with the present-day topography of the region. A similar approach was adopted for reverse-fault related anticlines^73^.

As comparison, we used the SRTM15 DEM^42^ since the 15 arcseconds resolution corresponds, at the latitude of the study area to ~ 400 m which is comparable to the resolution of the grid used in our numerical model.

We normalised both the DEM and the model results with respect to the average altitude value. We then subtracted the predicted model from the measured topography.

The differences in uplift patterns from these models will reveal the sensitivity of uplift to variations of stress field orientation (Supplementary Fig. 5).

## Supplementary Information

Supplementary Information 1.

Supplementary Information 2.

## Data Availability

All data generated or analysed during this study are included in this published article (and its Supplementary Information files). Faults geometries are stored in plain text gocad file. It can be open with: SKUA-GOCAD: This is the main software package used to build and maintain the CFM. SKUA-GOCAD software requires a license and is not freeware. The Petroleum Experts Move Suite: Move requires a license, but is available at no charge to qualifying academic institutions. Move can directly import gocad t-surf files and has tools for manipulating fault meshes. The SCEC VDO: The SCEC-VDO is a free 3D visualization software package that was designed by researchers and undergraduate interns at SCEC].
