# Supplementary material for: A validated geomechanical model for the strike-slip restraining bend in Lebanon
Source: Sci Rep. 2022 Nov 22;12:20071. doi: 10.1038/s41598-022-24718-0 (PMC9684496; doi:10.1038/s41598-022-24718-0)
Supplement: Supplementary file 2 — Supplementary Information 2. [file 41598_2022_24718_MOESM2_ESM.docx]

# Supplementary material


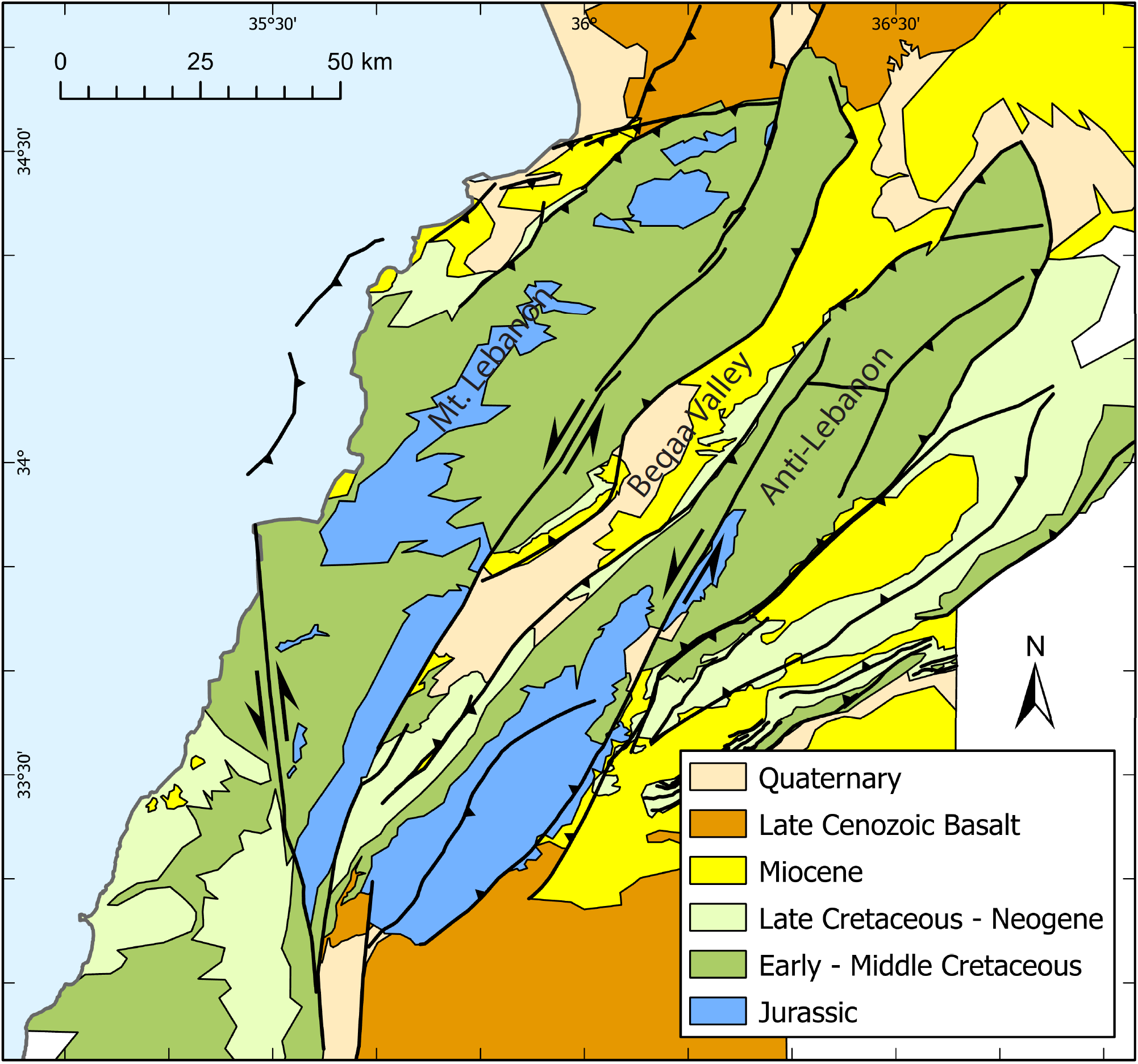


**Supplementary Figure 1 | Simplified geologic map of Lebanon LRB.** The map is redrawn from ^24^ for the Lebanon area and ^74^ for Syria. Unroofed areas exposing Jurassic and Cretaceous units highlight the domain with highest amount of erosion, caused by tectonic uplift. Map created in ^75^.


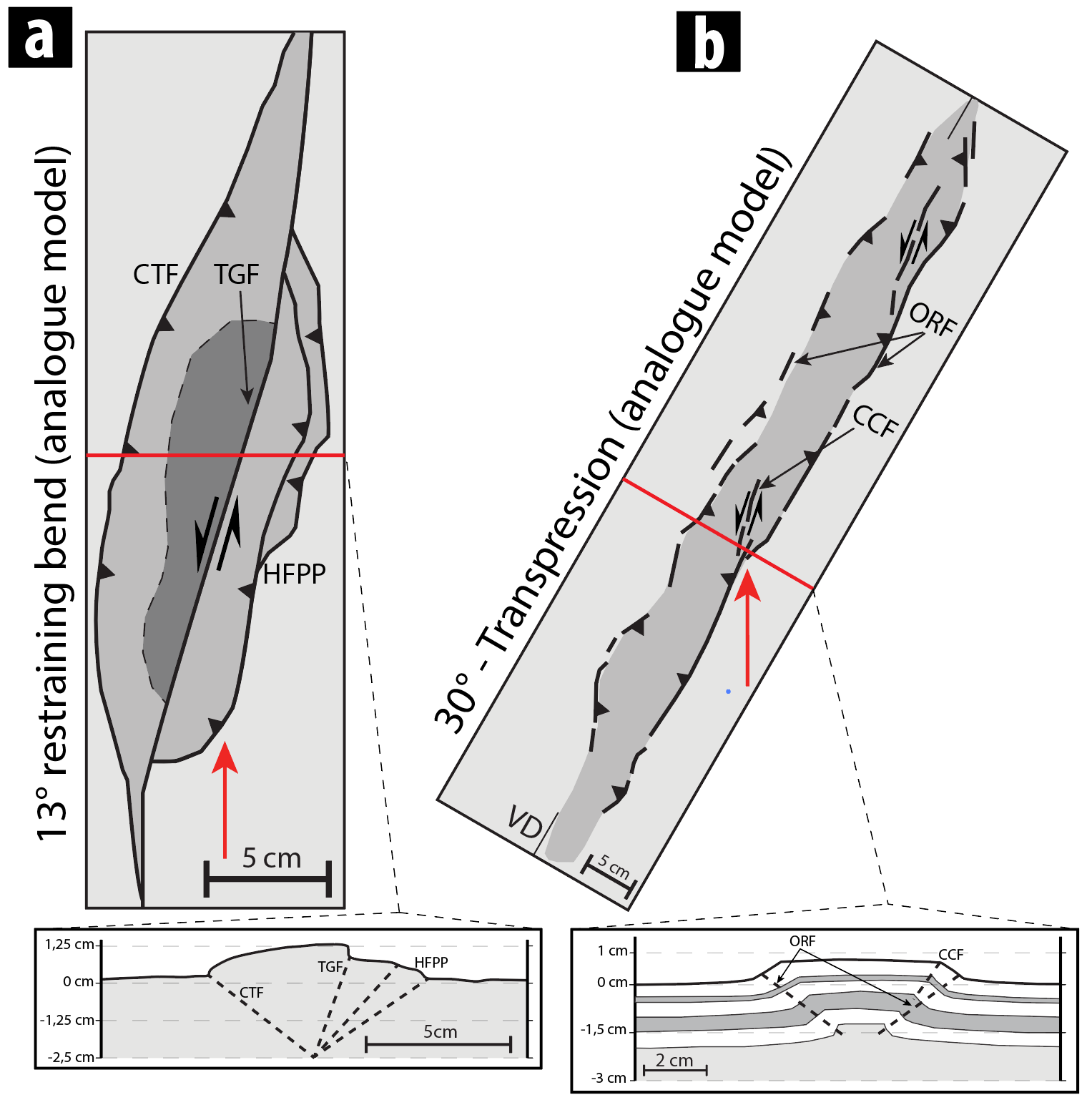


**Supplementary Figure 2 | Analogue models used for 3D fault geometry construction. a**, Model topography of plate displacement in the 13° restraining bend experiments and section view showing faults^51^. Three main faults are described: through going fault (TGF), curved thrust fault (CTF) and half pop-up structure (HFPP). **b**, Model topography of plate displacement in the 30° transpressional experiments along a straight velocity discontinuity (VD) and section view showing faults^47^. Two main faults are described: oblique reverse fault (ORF) and crosscutting fault (CCF). Red arrows indicate displacement of the moving plates.


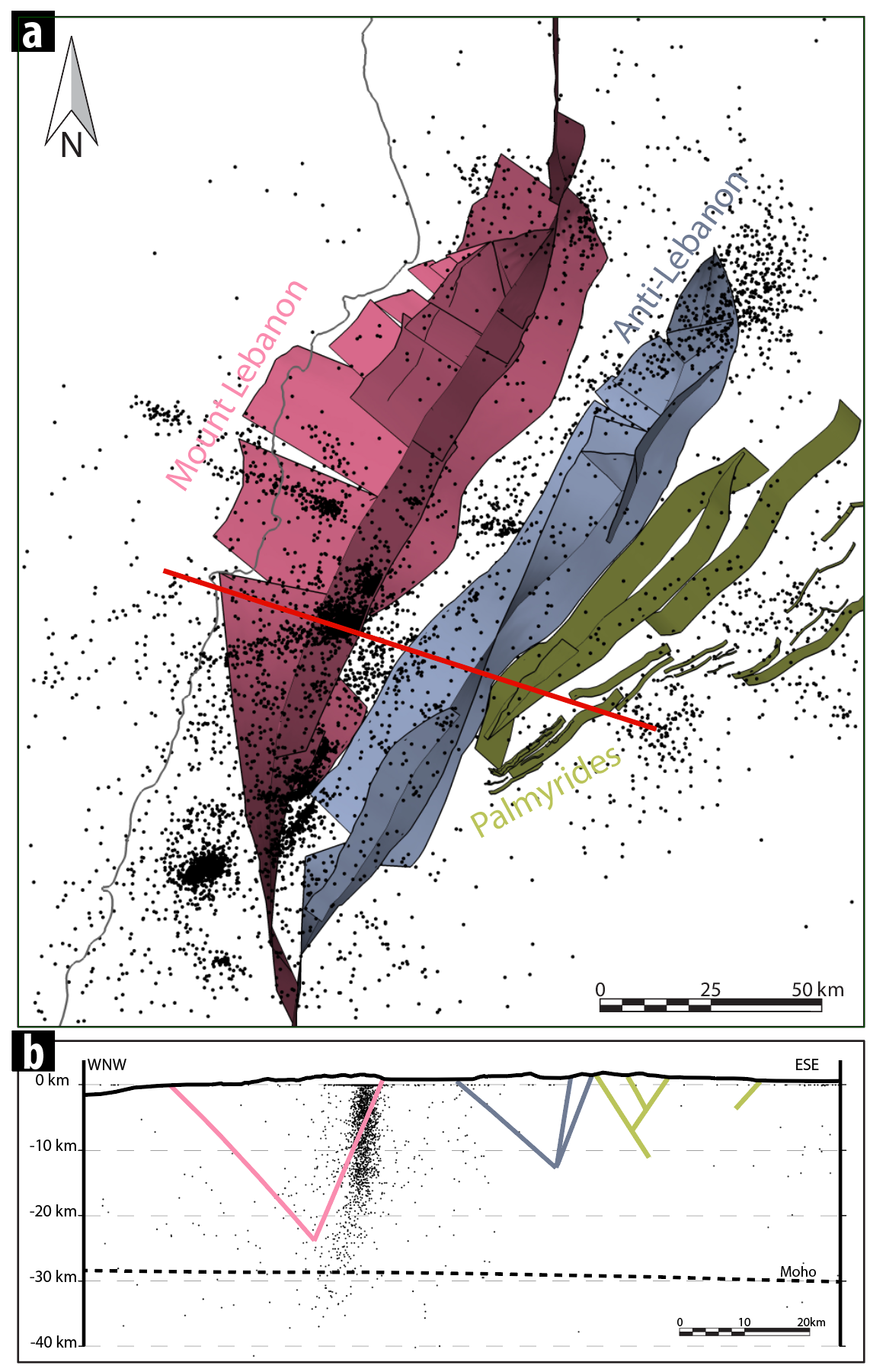


**Supplementary Figure 3 | Instrumental seismicity of the Lebanon Restraining Bend. a**, 3D fault geometry of faults and seismicity between 2006 and 2018 as recorded by GRAL. Data provided by CNRS Lebanon - National Center for Geophysical Research (http://www.cnrs.edu.lb/english/research/researchcenters/national-center-for-geophysics---download). **b**, Section view (location - red line in a.) showing seismicity (normal projection +/- 5km from the section) and faults intersection of our conceptual structural model. Seismicity reaches Moho depth and overlaps with the conceptual trace of Yammouneh fault.

###
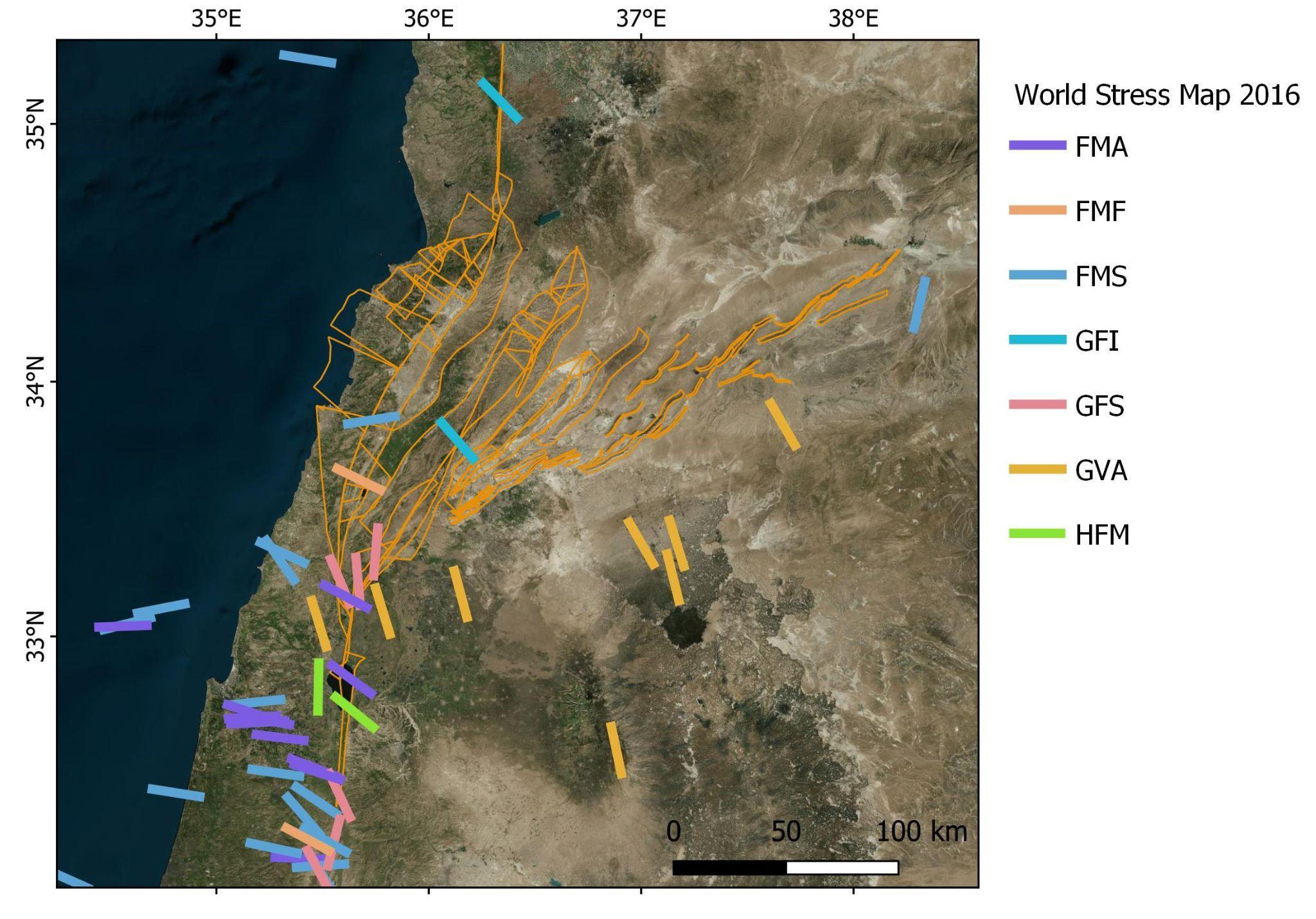


**Supplementary Figure 4 |** **Maximum horizontal stress orientation** from World Stress Map, 2016^64^ in the study area. FMA: average of p-axis or composite mechanism solutions; FMF: formal inversion of several focal mechanisms; FMS: single focal mechanism solution; GFI: inversion of fault-slip data observed on planes of a variety of trends; GFS: orientation from fault attitude and primary sense of offset; GVA: geologic-volcanic vent alignment; HFM: magnitude reported for maximum depth. Basemap of the stress figure is from Bing Aerial (https://www.bing.com/maps). Map created in QGIS 3.20 Odense ^76^.


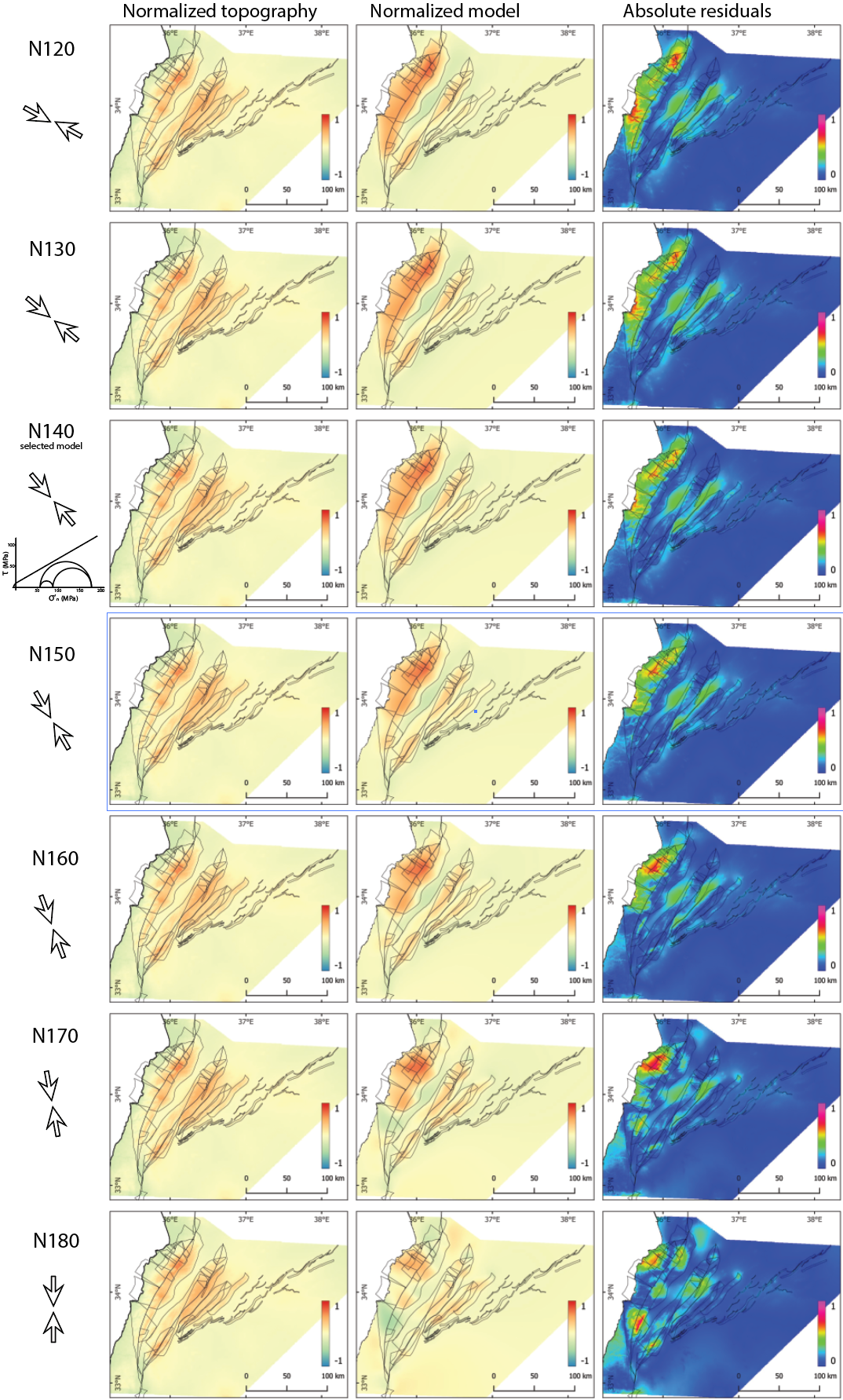


**Supplementary Figure 5 | σ1 strike variation results.** In total seven numerical models were performed where the strike of the σ1 was tested from N120 to N160 with an increment of 5°. Models ranging between N145 and N160 do not produce elevation change along the Roum fault which is not consistent with LRB. Models within the strike of the σ1 ranging between N120 and N135 produce important uplift along the Mt. Lebanon as most of the faults are almost perpendicular to maximum horizontal stress (σ1), therefore these faults are activated as compressional structures. The selected model (N140), which is presented in the paper in much more detail, produces continuous uplift along Mt. Lebanon, while all of its structures are activated as observed in nature, and the absolute residuals show in general very promising results. Dominant incostanceses between the model and the LRB can be explained by missing erosion and sedimentation in our calculations. Red arrows indicate a strike of maximum horizontal stress (σ1).

**Supplementary Table 1 | Model parameters.** Parameters used for all numerical models performed in this study. The three-dimensional boundary-element method, incorporated in Fault Response Modelling of MOVE software was used.

| Model parameters | |  |
| --- | --- | --- |
| Pressure profile depth | 5.75 | km |
| Average rock density | 2600 | kg/m^3^ |
| Water density | 1050 | kg/m^3^ |
| σ1 | 237.3 | MPa |
| σ2 | 146.5 | MPa |
| σ3 | 116.3 | MPa |
| Pore pressure | 59.2 | MPa |
| Poisson ratio | 0.25 |  |
| Young Modulus | 40 | GPa |
| Friction coefficient | 0.6 |  |
